# Soybean *GmMYB73* promotes lipid accumulation in transgenic plants

**DOI:** 10.1186/1471-2229-14-73

**Published:** 2014-03-24

**Authors:** Yun-Feng Liu, Qing-Tian Li, Xiang Lu, Qing-Xin Song, Sin-Man Lam, Wan-Ke Zhang, Biao Ma, Qing Lin, Wei-Qun Man, Wei-Guang Du, Guang-Hou Shui, Shou-Yi Chen, Jin-Song Zhang

**Affiliations:** 1 State Key Lab of Plant Genomics, Institute of Genetics and Developmental Biology, Chinese Academy of Sciences, Beijing 100101, China; 2 State Key Lab of Molecular Developmental Biology, Institute of Genetics and Developmental Biology, Chinese Academy of Sciences, Beijing 100101, China; 3 Institute of Soybean Research, Heilongjiang Provincial Academy of Agricultural Sciences, Harbin 150086, China

**Keywords:** Fatty acids, *GmMYB73*, Seed size, Soybean, Lipid, Thousand-seed weight

## Abstract

**Background:**

Soybean is one of the most important oil crops. The regulatory genes involved in oil accumulation are largely unclear. We initiated studies to identify genes that regulate this process.

**Results:**

One MYB-type gene *GmMYB73* was found to display differential expression in soybean seeds of different developing stages by microarray analysis and was further investigated for its functions in lipid accumulation. GmMYB73 is a small protein with single MYB repeat and has similarity to CPC-like MYB proteins from Arabidopsis. GmMYB73 interacted with GL3 and EGL3, and then suppressed *GL2*, a negative regulator of oil accumulation. *GmMYB73* overexpression enhanced lipid contents in both seeds and leaves of transgenic Arabidopsis plants. Seed length and thousand-seed weight were also promoted. *GmMYB73* introduction into the Arabidopsis *try cpc* double mutant rescued the total lipids, seed size and thousand-seed weight. *GmMYB73* also elevated lipid levels in seeds and leaves of transgenic Lotus, and in transgenic hairy roots of soybean plants. GmMYB73 promoted *PLDα1* expression, whose promoter can be bound and inhibited by GL2. *PLDα1* mutation reduced triacylglycerol levels mildly in seeds but significantly in leaves of Arabidopsis plants.

**Conclusions:**

GmMYB73 may reduce *GL2*, and then release GL2-inhibited *PLDα1* expression for lipid accumulation. Manipulation of *GmMYB73* may potentially improve oil production in legume crop plants.

## Background

As an important oil crop, soybean provides oils for edible, industrial and new energy uses to meet the increasing demand [1,2]. The oil content in soybean seeds generally ranges from 13% to 22% in various soybean cultivars, and is relatively low compared to most other oilseed crops [3]. High content of oil in soybean seeds is hence desirable and has been a major goal of breeding and genetic engineering.

The storage compounds of most seeds consist of carbohydrates, oils, and storage proteins and these compounds contribute up to 90% or more of the dry seed weight. Fatty acids are stored as triacylglycerols (TAGs) in seeds [4,5]. The regulation of TAG metabolism involves two mechanisms. One is short term regulation based on substrate availability, allosteric effectors and/or enzyme modification. Another way that regulates lipid biosynthesis is through control of enzyme synthesis and turnover rate. These have been achieved by direct modification of fatty acid biosynthesis enzyme to alter relative amounts of particular natural fatty acids, to produce novel fatty acid or to engineer the fatty acid chain length [6-9]. Several reports disclose that overexpression or modification of key enzymes, such as acetyl-CoA carboxylase (ACCase) and diglyceride acyltransferase (DGAT), alters seed oil accumulation [10-13].

In addition to the regulation at the key enzymes and major steps of lipid metabolism pathway, accumulation of fatty acids and lipids is also regulated at transcriptional level. A few transcription factors have been identified as master regulators of seed oil content by screens of Arabidopsis mutants, such as *LEC1, LEC2* and *WRI1*[14-17]. Manipulation of transcription factors can regulate expression of genes in fatty acid biosynthesis and alter the fatty acid/oil levels [18-23]. Other seed-specific transcription factors may also have roles in regulation of oil accumulation [24-26]. The storage lipid accumulation may be further regulated by new transcription factors, kinases/phosphatases and/or proteins involved in RNA regulation [27-31]. Additionally, new strategy has been developed to increase oil content by overproducing WRI1 for oil biosynthesis but reducing starch biosynthesis at the same time [32].

MYB proteins play important roles in multiple aspects of plant growth, development and responses to biotic and abiotic factors [33-36]. MYB proteins can be classified into three types: the R2R3-type MYB with two repeats, the R1R2R3-type MYB with three repeats and the third type usually containing single repeat or atypical repeat in plant. *CPC*-like *(CAPRICE)* gene family encodes small proteins with single MYB motif and negatively regulates trichome development in *Arabidopsis*. Seven CPC-like proteins have been identified in *Arabidopsis*, including *CPC*[37] and *TRY*[38]*.*

Previously, we have studied soybean transcription factors and analyzed their roles in abiotic stress tolerance [35,39-42]. Three *MYB* genes *GmMYB76*, *GmMYB92* and *GmMYB177* have been found to play differential roles in stress tolerance in transgenic plants [35]. We also found that two soybean transcription factor genes *GmDof4* and *GmDof11* enhance lipid content in seeds of transgenic Arabidopsis plants, through upregulation of lipid biosynthesis-related genes and downregulation of storage protein gene by direct binding to their promoter regions [43]. More factors may be involved in regulation of lipid biosynthesis in soybean plants.

In order to identify new genes that possibly regulate accumulation of fatty acids in developing seeds, microarray analysis was performed using RNAs from soybean developing seeds at different stages and a series of differentially expressed *MYB* genes were chosen and characterized for their functions [44]. Among the nine *MYB* genes analyzed, only *GmMYB73,* a gene encoding a protein with single MYB repeat, altered the lipid content in transgenic Arabidopsis seeds [44]. This gene was investigated in detail due to its potential ability to increase lipid levels. Overexpression of *GmMYB73* increased the oil content in both seeds and leaves of transgenic Arabidopsis and transgenic Lotus plants, and in transgenic hairy roots of soybean plants. These functions may be achieved through GmMYB73 interaction with GL3/EGL3, suppression of *GL2* and activation of *PLDα1*.

## Results

### *GmMYB73* gene expression

**Figure 1:**
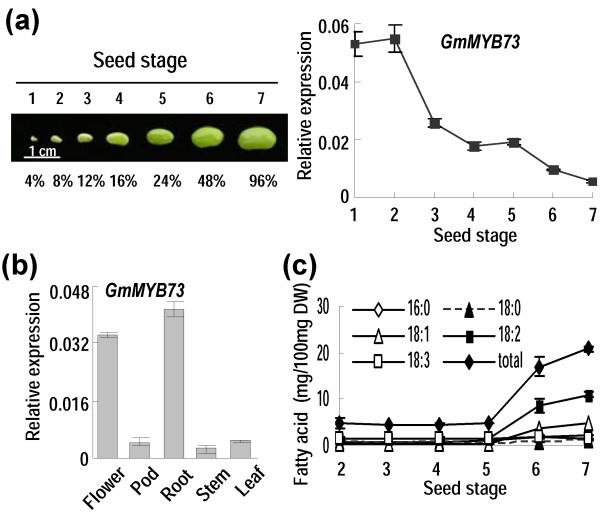
***GmMYB73 *expression. (a)***GmMYB73* expression at different stages of developing soybean seeds. Left panel: different stages of soybean seeds; percentages indicate relative seed weight compared to the full-sized seed without desiccation. Right panel: *GmMYB73* relative expression in seeds at different stages. **(b)***GmMYB73* expression in different organs of soybean plants. Stage five pods were used. Three-week-old seedlings were used for harvest of stem, root and leaf. For gene expression in **(a)** and **(b)**, error bars indicate SD (n = 4). **(c)** Fatty acid contents in seeds at different developmental stages. Total contents are derived from the content of five individual fatty acids. The values are in dry weight. Error bars indicate SD (n = 4).

The developing soybean seeds were divided into seven stages from pollination to mature seeds, and the relative seed weigh at each stage ranged from 4% to 96% when compared to the full size seeds without desiccation (Figure 1a, left panel). Expression of *GmMYB73* (DQ822927) encoding a protein of 73 residues with single MYB repeat was analyzed in seeds at these developmental stages by quantitative PCR. The *GmMYB73* gene was originally identified from microarray analysis using RNAs from developing seeds at different stages [44]. *GmMYB73* expression drastically decreased after stage two during seed development and reached the lowest level when seeds were near the full size (Figure 1a, right panel). The *GmMYB73* expression was also examined in different organs of soybean, and relatively higher expression levels were observed in flower and root but not in pod (stage five), stem or leaf tested (Figure 1b).

Fatty acid levels were measured in the developing soybean seeds and after stage five, the total fatty acid and composition changed significantly (Figure 1c).

### Phenotypes of the transgenic Arabidopsis plants overexpressing *GmMYB73*

To study *GmMYB73* functions, we generated the construct harboring *GmMYB73* controlled by CaMV 35S promoter in pPROK II vector and transformed this gene into Arabidopsis plants using Agrobacterium-mediated floral dip transformation method. *GmMYB73* expression was examined (Additional file 1) in homozygous transgenic lines (OE-1, 2, 5, 7, and 10) and plant phenotypes were investigated.

All the transgenic lines expressing *GmMYB73* showed almost no trichomes compared to Col-0 (Additional file 1), suggesting that *GmMYB73* inhibits trichome formation. A phylogenetic analysis was performed to compare the relationship of GmMYB73 with other Arabidopsis MYB proteins involved in trichome formation (Additional file 1). GmMYB73 was clustered with seven CAPRICE (CPC)-like proteins with single MYB repeat from Arabidopsis, including CPC [37], TRY [38], TCL1, TCL2, ETC1, ETC2 and CPL3. The *GmMYB73*-overexpressing line OE-5 was crossed with *try cpc* double mutant, which has the distinct clustered trichomes (Additional file 1) [45]. The trichome formation in *try cpc* mutant harboring the homozygous *GmMYB73* transgenes (*try cpc/GmMYB73*) was suppressed in both leaves and stems, similar to that in *GmMYB73-*overexpressing plants (Additional file 1). These results suggest that GmMYB73 is a homologue of CPC and TRY and is involved in trichome formation. GmMYB172 (DQ822946) and GmMYB363 (FJ555058) are close homologues of GmMYB73 in soybean (Additional file 1).

### GmMYB73 binds to GL3 and EGL3 and inhibits *GL2* expression

**Figure 2:**
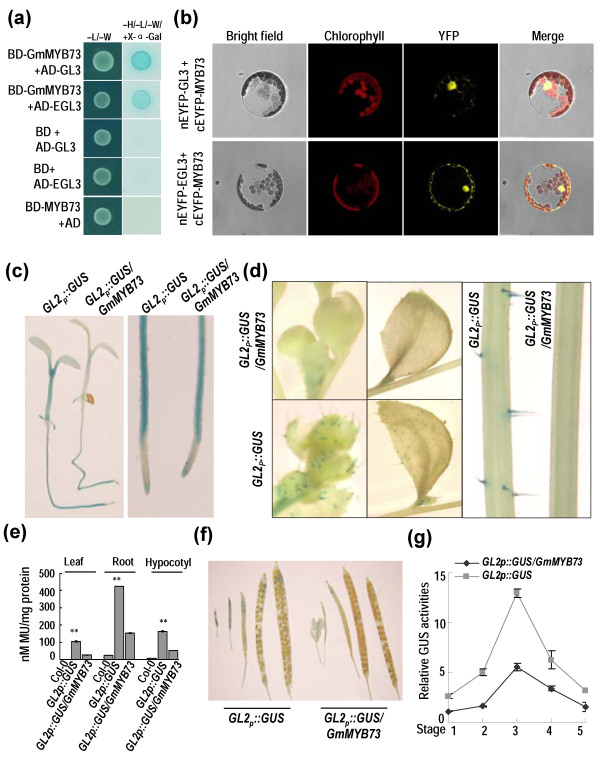
**GmMYB73 interacts with GL3 and EGL3, and inhibits *GL2 *expression. (a)** Interaction of GmMYB73 with GL3 and EGL3 in yeast two-hybrid assay. Yeast transformants were grown on control SD/–Leu/–Trp (left column), or selection medium SD/–Ade/–His/–Leu/–Trp with X-a-Gal and Aureobasidin A (right). Growth of cells and blue color on selection medium indicate positive interactions. Other combinations were used as negative controls. **(b)** BiFC was used to detect the interaction between GmMYB73 and GL3 or EGL3 in Arabidopsis protoplasts. Yellow fluorescence in YFP indicates positive interactions. **(c) ***GL2* promoter activity was inhibited by *GmMYB73* in aerial parts but partially suppressed in roots. Left: GUS staining in whole seedlings; right: GUS staining in roots. **(d)** cGmMYB73 suppressed *GL2* promoter activity and trichome formation on sepals of floral buds (left), leaves (middle) and stems (right). **(e)** *GL2* promoter activity was inhibited by GmMYB73 as revealed by GUS activity in leaves, roots and hypocotyls of transgenic plants. Asterisks ‘**’ indicate a significant difference from Col-0 levels (P < 0.01). **(f)** GmMYB73 inhibited *GL2* promoter activity during silique development as revealed from GUS staining. **(g)** GmMYB73 inhibited *GL2* promoter activity as revealed from relative GUS activity. GUS activity at stage 1 of *GL2p::GUS/GmMBY73* was set to 1 and all the other values were compared with it. The five stages corresponded to the silique phenotypes in **(f)** respectively.

In Arabidopsis, CPC*-*like R3 MYB proteins (e.g. TRY and CPC) are repressors for transcriptional activator and compete with GL1 (an R2R3-MYB factor) for binding to GL3 and EGL3, both are bHLH factors [46,47]. When bound to GL3 and EGL3, the formation of GL3/EGL3/GL1/TTG1 (a WD40 protein) transcription complex was blocked and thereby *GL2* transcription for a homeodomain transcription factor was inhibited. Finally the formation of trichome was repressed [38,46,47]. *GL2* mutation results in enhanced oil accumulation in seeds of Arabidopsis [18,23]. We also found that both GL3 and EGL3 could interact with GmMYB73 in yeast two-hybrid assay (Figure 2a). Furthermore, these interactions were confirmed in Arabidopsis protoplasts using BiFC assay, as revealed from yellow fluorescence in nucleus and cytoplasm (Figure 2b). These results indicate that GmMYB73 can interact with GL3 and EGL3.

To determine whether GmMYB73 affects *GL2* expression, transgenic Arabidopsis plants harboring *GL2 promoter::GUS* (*GL2*_*p*_*::GUS*) [48] were crossed with the transgenic plants overexpressing *GmMYB73* (OE-5) plants, and GUS staining was examined in homozygous *GL2*_*p*_*::GUS/GmMYB73* transgenic plants. In the *GL2*_*p*_*::GUS* plant*,* GUS staining was observed in hypocotyl, root, shoot apex and trichomes of flower buds, leaf and stem (Figure 2c, d). GUS staining was strongly inhibited by GmMYB73 in hypocotyl and shoot apex in *GL2*_*p*_*::GUS/GmMYB73* plants (Figure 2c). Trichomes and GUS staining were barely detectable in flower buds, leaf and stem of these plants (Figure 2d). GUS staining was only partially suppressed in roots of *GL2*_*p*_*::GUS/GmMYB73* plants (Figure 2c). GUS activity was also quantified in leaves, hypocotyls and roots of above transgenic plants, and the levels were consistent with the GUS staining results (Figure 2c, d, e). These results indicate that GmMYB73 negatively regulates trichome formation by interacting with GL3 and EGL3 to repress *GL2* transcription in transgenic Arabidopsis plants.

Siliques of five developmental stages in *GL2*_*p*_*::GUS* and *GL2*_*p*_*::GUS/GmMYB73* plants were examined and we found that GUS staining gradually decreased in developing siliques of *GL2*_*p*_*::GUS* plants (Figure 2f). *GmMYB73* partially suppressed GUS staining and GUS activity in siliques of *GL2*_*p*_*::GUS/GmMYB73* plants (Figure 2f, g). These results indicate that GmMYB73 inhibits *GL2* promoter activity in developing siliques.

### Overexpression of *GmMYB73* promotes seed size and thousand-seed weight

**Figure 3:**
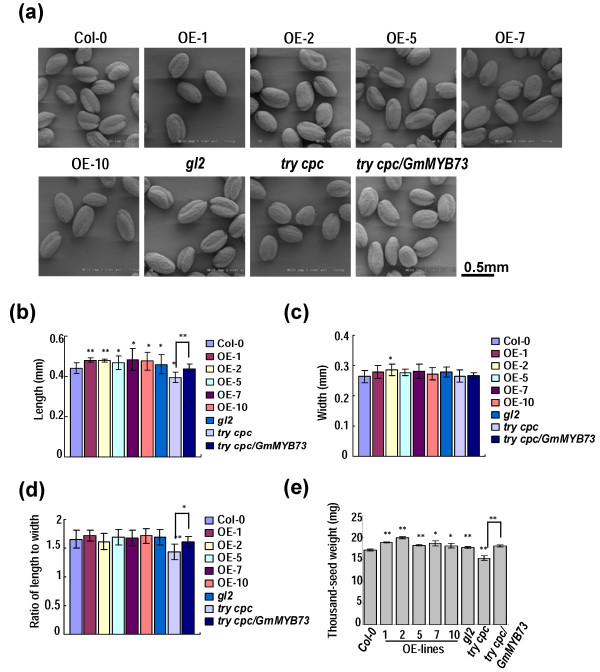
***GmMYB73 *overexpression controls seed size and thousand-seed weight of transgenic Arabidopsis plants. (a)** Morphology of Arabidopsis seeds under scanning electron microscope. Seeds from *GmMYB73*-overexpressing Arabidopsis plants (OE), *gl2-2*, *try cpc* and *try cpc/GmMYB73* lines were used. **(b)** Comparison of seed length from various plant seeds. Error bars indicate SD (n = 20 ~ 30). The values from *try cpc/GmMYB73* line are only compared with those from the *try cpc* mutant. ‘**’ and ‘*’ above the columns indicate a significant difference from Col-0 or between the compared pairs at P < 0.01 and P < 0.05, respectively. **(c)** Comparison of seed width from various A. thaliana lines. Others are as in **(b)**. **(d)** Comparison of the ratio of seed length to seed width. Others are as in **(b)**. **(e)** Comparison of thousand-seed weight in various A. thaliana lines. Error bars indicate SD (n = 4). Others are as in **(b)**.

*GmMYB73* expression changed at different stages of developing soybean seeds (Figure 1) and affected *GL2* expression in siliques of transgenic plants (Figure 2f, g). We then examined whether seed size was changed in *GmMYB73*-overexpressing plants. Under scanning electron microscope, we found that the *try cpc* double mutant had smaller seeds compared to Col-0 whereas the *GmMYB73*-transgenic plants and *gl2-2* mutant appeared to have larger seeds (Figure 3a). Further measurements of seed size revealed that seed length, but not seed width, was significantly increased in *GmMYB73*-overexpressing plants and *gl2* mutant compared to Col-0 (Figure 3b, c). The ratio of seed length to width was not significantly changed in these plants (Figure 3d). Seed length and ratio of length to width, but not seed width, was significantly reduced in seeds from *try cpc* double mutant compared to Col-0 (Figure 3b, c, d). Introduction of the *GmMYB73* into the double mutant *try cpc* rescued the seed length and ratio of length to width (Figure 3b, d). These results suggest that *GmMYB73* and possibly its homologues *TRY* and *CPC* affect seed development and seed size.

Thousand-seed weights were also measured and the five *GmMYB73*-overexpressing lines had significantly higher thousand-seed weights than Col-0 (Figure 3e). The *gl2* seeds also had slightly but significantly higher levels of the parameter (Figure 3e). In contrast, *try cpc* double mutant had significantly lower thousand-seed weight than Col and introduction of *GmMYB73* recovered the level in *try cpc/GmMYB73* plants (Figure 3e). These results indicate that GmMYB73, its homologues TRY and CPC, and GL2 regulate seed development.

### *GmMYB73* increases lipid contents in seeds and leaves of transgenic Arabidopsis and Lotus plants, and in transgenic hairy roots of soybean plants

**Figure 4:**
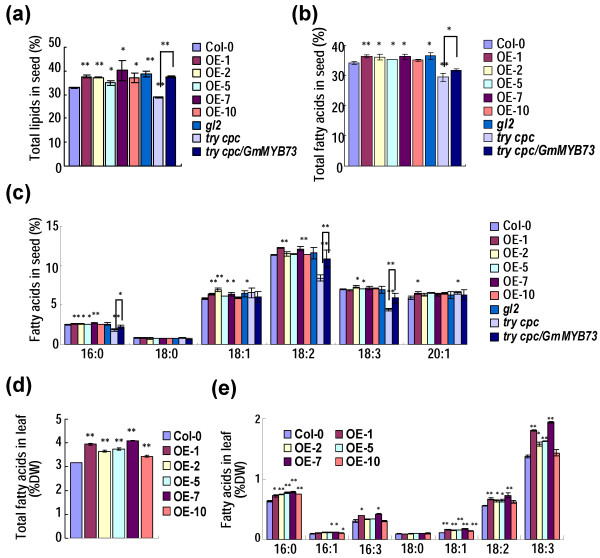
***GmMYB73 *increases lipid contents in seeds and leaves of transgenic Arabidopsis plants. (a)** Total lipid contents in seeds of Col, *GmMYB73*-transgenic plants (OE-1, 2, 5, 7, 10) *gl2-2*, *try cpc*, and *try cpc/GmMYB73*. Error bars indicate SD (n = 4) and the values are in dry weight for seeds. The values from *try cpc/GmMYB73* line are only compared with those from the *try cpc* mutant. Asterisks indicate a significant difference from Col-0 or between the compared pairs (*P < 0.05 and **P < 0.01). **(b)** Contents of total fatty acids in seeds of various plants. Error bars indicate SD (n = 4). Others are as in **(a)**. **(c)** Compositions of fatty acids in seeds of various plants. Error bars indicate SD (n = 4). Others are as in **(a)**. **(d)** Total fatty acids in plant leaves. Error bars indicate SD (n = 4) and the values are in dry weight. Others are as in **(a)**. **(e)** Compositions of fatty acids in plant leaves. Error bars indicate SD (n = 4) and the values are in dry weight. Others are as in **(a)**.

Total lipid content in seeds of Col-0, *GmMYB73*-overexpressing lines (OE-1, -2, -5, -7 and -10), and various mutant lines was measured. All five *GmMYB73*-transgenic lines and *gl2* mutant had higher levels of total lipids than Col-0, and the increase ranged from 5.9% to 17.9% (Figure 4a). The *try cpc* mutant had lower lipid content than Col-0 and the *GmMYB73* transformation increased the lipid content in *try cpc/GmMYB73* plants compared to the double mutant (Figure 4a). Contents of total fatty acids in *GmMYB73-*transgenic lines and *gl2* mutant were also increased compared to Col-0 (Figure 4b). The total fatty acids in *try cpc* mutant were reduced and introduction of *GmMYB73* in the mutant largely recovered total fatty acid content to the WT level (Figure 4b). As to fatty acid compositions, except 18:0, other fatty acids showed slight increase or no significant change in *GmMYB73*-transgenic lines and *gl2* mutant compared to Col-0 (Figure 4c). In *try cpc* mutant seeds, levels of three fatty acids (16:0, 18:2, 18:3) were significantly reduced compared to Col-0 and partially rescued by *GmMYB73* expression (Figure 4c).

Fatty acid levels were measured in leaves of various plant lines. *GmMYB73*-overexpressing lines had significantly higher total fatty acid contents in leaf compared to Col-0 (Figure 4d). Level of each fatty acid compostion, except 18:0, was also significantly or slightly increased in the transgenic lines compared to the levels in Col-0 (Figure 4e). These results indicate that *GmMYB73* increased contents of total lipids and total fatty acids in seeds and leaves of transgenic Arabidopsis plants.

**Figure 5:**
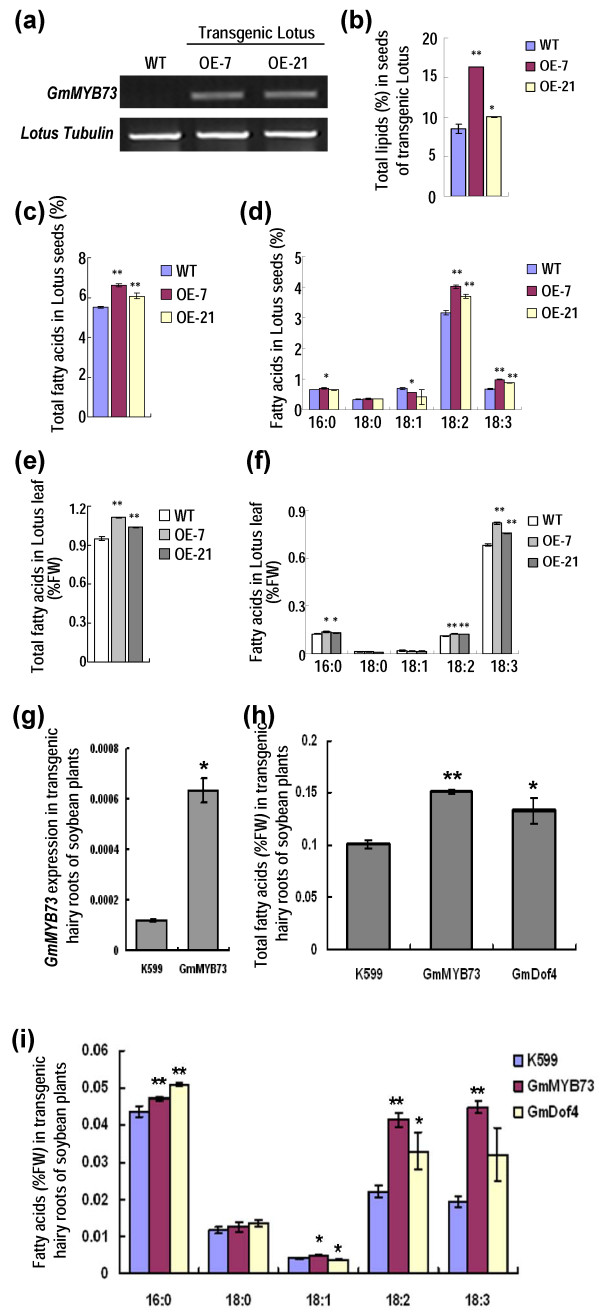
***GmMYB73 *enhances lipid contents in seeds and leaves of transgenic Lotus plants, and in transgenic hairy roots of soybean plants. (a)***GmMYB73* expression in leaves of transgenic Lotus plants. Two lines OE-7 and OE-21 were used. Tubulin gene was amplified as a control. **(b)** Contents of total lipids in transgenic seeds compared to WT. Error bars indicate SD (n = 4) and the values are in dry weight. Asterisks indicate significant difference compared to WT (**P < 0.01, *P < 0.05). **(c)** Contents of total fatty acids in transgenic seeds. Error bars indicate SD (n = 4) and others are as in **(b)**. **(d)** Contents of each fatty acid composition in transgenic seeds. Others are as in **(b)**. **(e)** Contents of total fatty acids in leaves of transgenic Lotus plants. The values are in fresh weight. Others are as in (b). **(f)** Contents of each fatty acid in leaves of transgenic Lotus plants. The values are in fresh weight. Others are as in **(b)**. **(g)***GmMYB73* expression in transgenic hairy roots of soybean plants. K599: control roots. GmMYB73: *GmMYB73*-transgenic hairy roots. Error bars indicate SD (n = 4). Asterisk indicates significant difference compared to control K599 (*P < 0.05). **(h)** Total fatty acid levels in *GmMYB73*-transgenic hairy roots. K599: control roots. GmMYB73: *GmMYB73*-transgenic hairy roots. GmDof4: *GmDof4*-transgenic hairy roots as a positive control for lipid accumulation. Error bars indicate SD (n = 4). The values are in fresh weight. Asterisks indicate significant difference compared to control K599 (**P < 0.01, *P < 0.05). **(i)** Levels of each fatty acid composition in *GmMYB73*-transgenic hairy roots. The values are in fresh weight. Other indications are as in **(h)**.

Soybean is a legume plant and we further transformed the *GmMYB73* into the legume Lotus japonicus (Leo) plants. Two transgenic lines were identified and both displayed *GmMYB73* expressions compared to WT (Figure 5a). Total lipids and total fatty acids in seeds of the two lines were significantly increased compared to WT plants (Figure 5b, c). As for the fatty acid composition, only two fatty acids (18:2 and 18:3) showed significant increase in the transgenic seeds (Figure 5d). Fatty acid levels in leaves of the transgenic plants were also determined and we found that total fatty acids and three fatty acids (16:0, 18:2 and 18:3) were apparently enhanced (Figure 5e, f). These results indicate that *GmMYB73* increases total lipid and total fatty acid contents in seeds and leaves of Lotus transgenic plants.

Considering that *GmMYB73* enhanced lipid contents in both seeds and leaves of transgenic Arabidopsis and Lotus plants, we further examined whether GmMYB73 could elevate lipid accumulation in transgenic hairy roots of soybean plants. The *GmMYB73*-overexpressing vector was transfected into Agrobacterium rhizogenes strain K599 and the bacterium was used to infect hypocotyls of soybean seedlings through injection. *GmMYB73* expression was much higher in *GmMYB73*-transgenic hairy roots (GmMYB73) than K599-regenerated roots (K599) (Figure 5g). Levels of total fatty acids and each of the three fatty acids (16:0, 18:2 and 18:3) exhibited apparent increase in *GmMYB73*-transgenic hairy roots compared to K599 control roots (Figure 5h, i). *GmDof4,* a gene that enhanced lipid levels in transgenic Arabidopsis plants in our previous study [43], can also increase the fatty acid content in the transgenic hairy roots of soybean plants (Figure 5h, i).

### GmMYB73 enhances expression of *PLDα1* whose promoter can be bound by GL2

**Figure 6:**
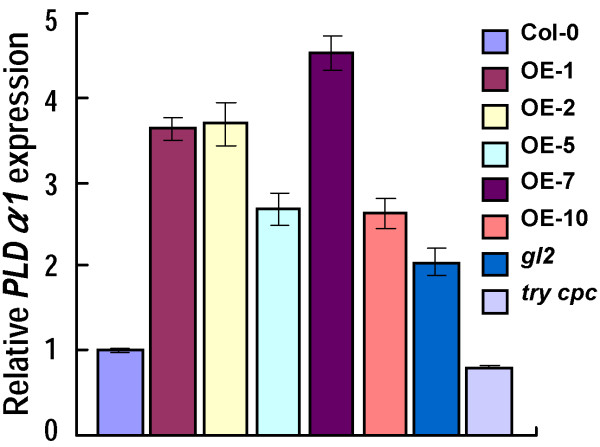
**GmMYB73 promotes *PLDα1 *expression.** Expression of *PLDα1* in leaves of *GmMYB73*-overexpressing Arabidopsis plants (OE-1, 2, 5, 7, 10), *gl2-2*, *try cpc* and wild type Col-0. Error bars indicate SD (n = 4).

GmMYB73 inhibited *GL2* expression (Figure 2). Seeds of *GmMYB73*-overexpressing plants and *gl2* mutant accumulated more lipids and fatty acids (Figure 4). We examined whether GmMYB73 altered downstream gene expressions through suppression of *GL2*. Mutation of GL2 results in an increase in seed oil contents [18]. GL2 represses phospholipase D ζ1 (*PLDZ1*) gene expression [49]. PLDs affect lipid composition [50-52]. However, increase of seed oil content in *gl2* mutant is not due to *PLDZ1* or *PLDZ2* expression [23]. We found that *PLDα1* was up-regulated in leaves of *GmMYB73*-overexpressing plants and *gl2* mutant*,* but down-regulated in *try cpc* plants compared to Col-0 (Figure 6). These results imply that the GmMYB73 may suppress expression of *GL2*, and thus activate *PLDα1* expression.

**Figure 7:**
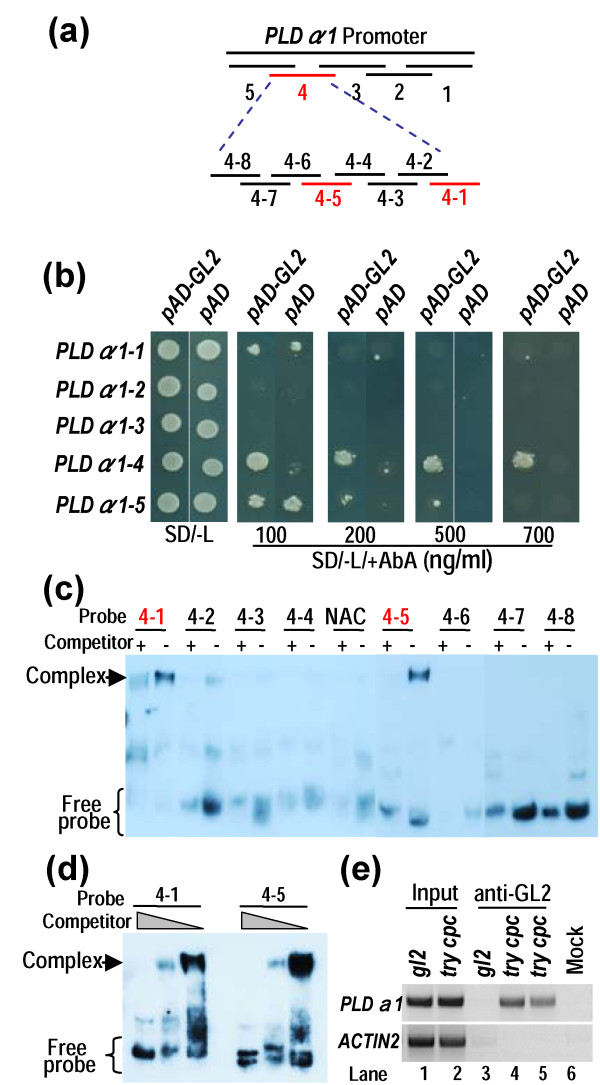
**GL2 bound to the *PLDα1 *promoter. (a) **Diagram of *PLDα1* promoter region. The promoter was divided into five fragments. The fourth fragment was further divided into eight small fragments. Fragments in red indicate binding by GL2. **(b)** GL2 bound to *PLDα1-4* region. Different regions of *PLDα1* promoter were cloned into pAbAi vector and these plasmids were co-transfected into yeast Y1HGold cells with pAD-GL2. Growth of transfected yeast cells on AbA medium indicates binding of GL2 to the corresponding elements. **(c)** Gel shift assay to test GL2 binding of 4–1 and 4–5 segments in *PLDα1* promoter. Proteins were incubated with labeled probes in the presence (+) or absence (-) of 200-fold molar excess of unlabelled competitors. A NAC binding sequence was added as a negative control. Arrow indicates protein/DNA complex. **(d)** GL2 binding of 4–1 and 4–5 segments in *PLDα1* promoter in the presence of labeled probes plus 500-fold, 200-fold and 0-fold molar excess of unlabelled competitors (from left to right lane respectively). **(e)** GL2 binding to *PLDα1-4* by Chromatin immunoprecipitation (ChIP) assay. ChIP was performed with *try cpc* (root in lane 4 and leaf in lane 5) and *gl2-2* plants (lane 3) using anti-GL2 antibody. Primer sets specific for the region of *PLDα1-4* were used in PCR reactions. *ACTIN2* was amplified as a control. Sonicated chromatin with incubation of second antibody (anti-mouse IgG) was used as a mock control (lane 6). The supernatant of sonicated chromatin from *gl2-2* and *try cpc* were used as input control (lane 1 and lane 2).

We further studied whether *PLDα1* promoter can be bound by GL2 in yeast one-hybrid assay. Five overlapping DNA fragments (1: -1 to -263; 2: -246 to -492; 3: -472 to -753; 4: -739 to -1038; 5: -1021 to -1328) in *PLDα1* promoter were tested for GL2 binding (Figure 7a). Only fragment *PLDα1-4* was bound by GL2 as revealed from growth of yeast transformants harboring pAD-GL2 and pAbAi-*PLDα1-4* in selection medium SD/-Leu/+AbA (Figure 7b). *PLDα1-4* was further divided into eight regions (Figure 7c; Additional file 2), and these small regions were further tested for GL2 binding using a gel shift analysis. GL2 bound specifically to two regions of *PLDα1-4* (4–1 and 4–5) (Figure 7c). Increasing concentrations of the non-labeled competitors significantly reduced the band intensity of the DNA-protein complexes, indicating that the GL2 binding to these elements was specific (Figure 7d). A NAC protein binding element (NAC) was used as a negative control for GL2 binding (Figure 7c). These results indicate that GL2 specifically binds to two elements in *PLDα1* promoter regions.

Chromatin immunoprecipitation (ChIP) assay was used to determine the interaction of GL2 with *PLDα1* promoter directly. Specific primers were used to amplify *PLDα1-4*-1 and *PLDα1-4*-5 fragments and the fragments were confirmed by sequencing. GL2 bound to the promoter region of *PLDα1* in *try cpc* double mutant; however, no GL2/*PLDα1* promoter complex was detected in *gl2* mutant (Figure 7e). *ACTIN2* was used as a negative control. The ChIP results were negatively correlated with *PLDα1* expression (Figures 6 and 7e). These results indicate that GL2 binds to *PLDα1* promoter and negatively regulates *PLDα1* expression*.*

### *PLDα1* mutation affects lipid accumulation

**Figure 8:**
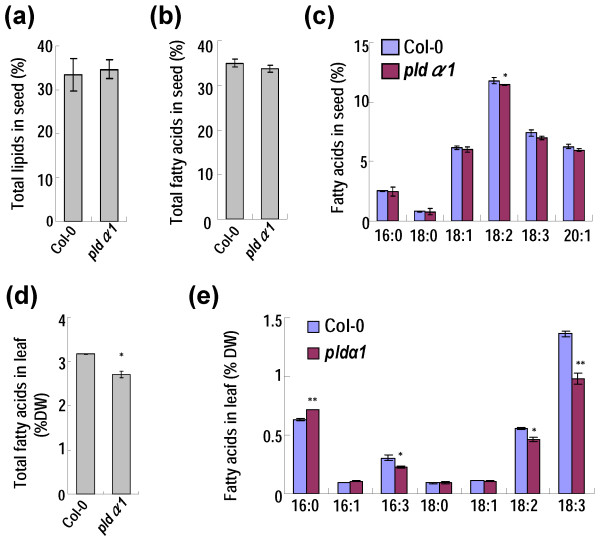
**Effect of *PLDα1 *deficiency on lipid and fatty acid accumulation. (a)** Total lipid contents in seeds of Col-0 and *pldα1* mutant plants. The values are in dry weight for seeds. **(b)** Contents of total fatty acids in seeds of Col-0 and *pldα1* plants. **(c)** Compositions of fatty acids in seeds of Col-0 and *pldα1* plants. **(d)** Total fatty acids in plant leaves. The values are in dry weight. **(e)** Compositions of fatty acids in plant leaves. The values are in dry weight. For **(a)** to **(e)**, error bars indicate SD (n = 4). Asterisks indicate a significant difference compared to the Col-0 controls (*P < 0.05 and **P < 0.01).

Since *PLDα1* expression was enhanced in *GmMYB73*-transgenic Arabidopsis plants, we examined whether *PLDα1* mutation would affect lipid accumulation. The total lipid content was not significantly changed in seeds of Arabidopsis *pldα1* mutant compared to Col-0 (Figure 8a). The total fatty acid content and linoleic acid level were slightly reduced in seeds of the mutant compared to Col-0 (Figure 8b, c). In leaves, the contents of total fatty acids and three compositions (16:3, 18:2 and 18:3) were significantly reduced in mutant compared to the Col-0 (Figure 8d, e).

We further investigated changes of lipid species in various *GmMYB73*-transgenic Arabidopsis plants, *pldα1* and other mutants. In seeds, compared to Col-0, the triacylglycerol (TAG) levels were substantially enhanced in *GmMYB73*-transgenic lines (OE-1, 2, 5, 7, 10), *gl2*, and *try cpc/GmMYB73* plants, but slightly reduced in *try cpc* and *pldα1* mutants (Table 1). The diacylglycerol (DAG) levels were significantly increased in *GmMYB73*-transgenic lines, *gl2*, and *try cpc/GmMYB73* plants. The phosphatidylcholine (PC) levels were somewhat reduced in *GmMYB73*-transgenic lines and *gl2.* The phosphatidic acid (PA) levels were not significantly changed in all the plants compared (Table 1). In leaf, the TAG, DAG and PA levels were mildly increased on average in *GmMYB73*-transgenic plants whereas the PC levels were slightly reduced in these plants (Table 2). In *pldα1* mutant leaf, the TAG and PA levels were significantly reduced whereas the PC level was slightly increased compared to that in Col-0 (Table 2). These results indicate that GmMYB73, GL2 and PLDα1 most likely act in the same pathway to regulate lipid accumulation.

**Table 1:** **Levels of TAG, DAG, PA and PC in seeds of Col-0, *GmMYB73*-transgenic Arabidopsis plants (OE-1, 2, 5, 7, 10), *gl2-2*, *try cpc*, *try cpc/GmMYB73 *** **and *pldα1***

	**Triacylglycerol (TAG)**	**Diacylglycerol (DAG)**	**Phosphatidic acid (PA)**	**Phosphatidylcholine (PC)**
Col-0	117.92 ± 6.64	0.19 ± 0.01	0.45 ± 0.04	6.36 ± 0.09
OE-1	148.68 ± 2.84**	0.26 ± 0.02**	0.46 ± 0.01	6.34 ± 0.03
OE-2	127.64 ± 2.88	0.22 ± 0.01*	0.45 ± 0.01	6.02 ± 0.20
OE-5	131.68 ± 2.66*	0.22 ± 0.01*	0.46 ± 0.01	5.97 ± 0.20*
OE-7	128.92 ± 2.46	0.26 ± 0.01**	0.45 ± 0.02	5.89 ± 0.29
OE-10	127.68 ± 0.54	0.25 ± 0.02*	0.45 ± 0.01	5.48 ± 0.31
*gl2*	142.76 ± 4.97*	0.30 ± 0.01**	0.46 ± 0.02	5.89 ± 0.16*
*try cpc*	110.59 ± 1.01	0.17 ± 0.02	0.48 ± 0.10	6.45 ± 0.04
*try cpc*/*GmMYB73*	140.80 ± 3.66*	0.21 ± 0.01*	0.51 ± 0.02	6.41 ± 0.30
*pldα1*	106.20 ± 7.97	0.20 ± 0.01	0.46 ± 0.01	6.53 ± 0.23

*Significant difference at P < 0.05 compared to the Col-0 value.

**Significant difference at P < 0.01 compared to the Col-0 value.

Each value (nmol/mgDW) is mean ± SD (n = 3).

**Table 2:** **Levels of TAG, DAG, PA and PC in leaves of Col-0, *GmMYB73*-transgenic plants (OE-1, 2, 5, 7, 10) and *pldα1***

	**Triacylglycerol (TAG)**	**Diacylglycerol (DAG)**	**Phosphatidic acid (PA)**	**Phosphatidylcholine (PC)**
Col-0	0.081 ± 0.005	0.046 ± 0.002	1.009 ± 0.090	10.874 ± 1.739
OE-1	0.116 ± 0.007*	0.059 ± 0.002	1.620 ± 0.090*	9.885 ± 1.150
OE-2	0.095 ± 0.007	0.064 ± 0.006	1.284 ± 0.111	8.202 ± 1.063
OE-5	0.106 ± 0.001*	0.070 ± 0.018	1.348 ± 0.139	7.207 ± 0.240
OE-7	0.117 ± 0.015*	0.072 ± 0.012*	1.322 ± 0.092	8.285 ± 1.042
OE-10	0.090 ± 0.019	0.057 ± 0.007	1.227 ± 0.014	8.899 ± 1.127
*pldα1*	0.062 ± 0.002*	0.046 ± 0.001	0.578 ± 0.004*	11.920 ± 0.429

*Significant difference at P < 0.05 compared to the Col-0 value.

Each value (nmol/mgDW) is mean ± SD (n = 3).

## Discussion

*GmMYB73* gene was identified to have variable expression levels during soybean seed development and to promote lipid accumulation in transgenic Arabidopsis. The effect of GmMYB73 on lipid accumulation may be achieved through reduction of GL2 expression, a negative regulator of oil accumulation, and then release of GL2-inhibited *PLDα1* expression.

Overexpression of *GmMYB73* enhanced seed size and thousand-seed-weight of Arabidopsis transgenic plants (Figure 3e). The *GmMYB73* also rescued the seed phenotype and thousand-seed weight in *try cpc* double mutant (Figure 3). These results indicate that *GmMYB73* or its homologues in Arabidopsis regulates seed development in addition to control of trichome formation. Several other genes involved in regulation of trichome formation also affect seed development. These include *TRANSPARENT TESTA GLABRA2*[53] and *TRANSPARENT TESTA GLABRA1*[54] in seed coat differentiation; *gl2* mutants do not produce releasable mucilage [23]; *GL3*, *EGL3*, and *TTG1* regulate seed coat proanthocyanidin biosynthesis [55,56]. Some of these genes also play roles in seed size control and thousand-seed weight regulation [57]. It should be mentioned that *gl2-1* seeds (Ler ecotype) show no change in thousand-seed weight when compared to WT [57]. In contrast, the present *gl2-2* (Col ecotype) exhibited slight but significant increase in thousand-seed weight when compared to control (Figure 3e). This discrepancy may be derived from different ecotypes used and/or different storage time for seeds. There are other genes that regulate both seed development and aspects of plant growth and development. Inhibition of ethylene receptor gene *OsETR2* expression in rice leads to increased thousand-grain weight and early flowering [58]. Mutation of *MHZ7/OsEIN2* affects grain shape and senescence [59]. Ethylene and salt-induced NIMA-related kinase NEK6 enhances plant growth and seed yield in Arabidopsis; however, thousand-seed weight and seed width are reduced [60]. Recently, two *DOF* genes *DOF4.2* and *DOF4.4* have been found to promote shoot branching but affect seed/silique development in Arabidopsis [61]. Other genes involved in regulating seed size and/or development are reviewed in [62].

*GmMYB73* overexpression promotes fatty acid accumulation in transgenic Arabidopsis and transgenic Lotus plants (Figures 4 and 5). The gene also fully rescued the total lipid level and partially rescued the total fatty acid level in *try cpc* double mutant (Figure 4), indicating that the *GmMYB73* is involved in upregulation of fatty acid accumulation. It should be noted that the increase of total fatty acid levels in seeds of the *GmMYB73*-transgenic Arabidopsis plants was likely not due to the increase in any specific fatty acid but rather due to the overall increase in each fatty acid level (Figure 4b, c). However, in leaves of the transgenic plants, the increase of the total fatty acids was due to an increase in linolenic acid (18:3) (Figure 4d, e). The difference in the accumulation patterns of fatty acids in seed and leaf tissues may be due the physiological and metabolic difference of these organs, where seed is a sink organ, while the leaf is a source organ. In *GmMYB73*-transgenic Lotus plants, the increase of total fatty acids in leaves is mainly due to increase of linolenic acid (18:3) (Figure 5f), similar to the case in leaves of Arabidopsis transgenic plants (Figure 4e). However, the seeds of transgenic Lotus plants showed a different change in fatty acid compositions compared to seeds of transgenic Arabidopsis, and the increase of total fatty acids was attributed to an increase in linolic acid (18:2) and/or linolenic acid (18:3) (Figure 5). The different accumulation patterns of fatty acid compositions in seeds of transgenic Arabidopsis and Lotus plants suggest that some different mechanisms may be affected in lipid accumulation in these plant species.

Due to the difficulty of soybean plant transformation, we adopted a method for generating transgenic hairy roots in soybean. *GmMYB73* overexpression in soybean transgenic hairy roots increased the total fatty acid levels and this elevation was likely due to the increase in linolic acid (18:2) and linolenic acid (18:3) levels (Figure 5h, i). It should be mentioned that the changes in fatty acid composition in soybean transgenic hairy roots were very similar to those in leaves of transgenic Arabidopsis plants (Figures 4e and 5i), suggesting that similar mechanisms may have been affected in vegetative organs of Arabidopsis and soybean. It is possible that *GmMYB73* would promote lipid accumulation in soybean seeds as it did in Arabidopsis seeds although further studies are needed to confirm this.

GmMYB73 reduced *GL2* expression (Figure 2), and GL2 has been found to be involved in oil accumulation in seeds. Shen *et al*. [18] reported that mutation in *GL2* gene led to increase in seed oil content compared to wild type levels. Overexpression of *BnaC.GL2*.b in Arabidopsis affected the seed oil accumulation [63]. More recently, Shi *et al*. [23] found that the *PLDZ1/2* genes, targets of GL2 [49], are not involved in seed oil accumulation. However, blocking *MUM4,* another downstream target of GL2, was found to reduce mucilage biosynthesis, which may be linked to an increase in seed oil production [23]. Presently, we found that *GmMYB73*-overexpressing plants and Arabidopsis *gl2-2* mutant had higher levels of *PLDα1*, due to inhibition of *GL2* expression by GmMYB73 (Figures 2 and 6). GL2 has been shown to bind to *PLDα1* promoter and inhibit *PLDα1* expression (Figure 7). Therefore, GmMYB73 may promote lipid accumulation by suppressing *GL2* expression, and thus enhancing *PLDα1* expression.

PLD hydrolyses the P-O bond of phosphatidylcholine (PC) to produce phosphatidic acid (PA) and choline. The PA is converted to 1,2-sn-diacylglycerol (DAG) [64] by the action of PA phosphatase and then DAG can be acylated to produce triacylglycerol (TAG). In developing soybean seeds, the major pathway for TAG formation is through conversion of PC to DAG and acylation of DAG to produce TAG [65]. Recently, Lee *et al*. [51,52] reported that the lipid profile was changed by suppression of *PLDα* in the soybean seeds and the total lipids and TAG signals tended to decrease in fresh seeds of *PLDα-*knockdown soybean. Presently, we find that *PLDα1* mutation in Arabidopsis substantially reduced the TAG levels in both seeds and leaves (Tables 1 and 2). In contrast, *GmMYB73*-transgenic Arabidopsis plants substantially had more TAG in seeds and leaves compared to Col-0 (Tables 1 and 2). It is noted that in seeds, most of the lipid are TAG, whereas in leaves, the major lipid species are PC and PA (Tables 1 and 2). The roughly negative correlation between PA and PC contents in leaves of both *GmMYB73*-transgenic plants and *pldα1* mutant (Tables 1 and 2) suggests that GmMYB73 may finally promote PA and possible DAG and TAG accumulation through PC conversion by PLDα1 function. Similar case may happen in *GmMYB73*-transgenic seeds although PA levels were not high, possibly due to an efficient conversion to DAG and TAG. All these data suggest that GmMYB73 may enhance lipid accumulation at least partially through repression of *GL2* and promotion of *PLDα1*. Other downstream genes (e.g., *MUM4*) may also be involved in this process [23]. It should be mentioned that the *GmMYB73*-overexpresing Arabidopsis plants have no or very little trichomes. Considering that trichome contained a multitude of wax components and can secrete lipids [66], the lack of trichome may save total energy and resources for lipid biosynthesis in other organs such as seeds and leaves of transgenic plants. Recently, *PLDα1* overexpression has been found to improve drought tolerance and increase seed yield [67], lending support to our present study.

It is interesting to note that, although GmMYB73 enhances lipid contents in transgenic seeds, its expression during soybean seed development is not consistent with the fatty acid accumulation pattern at these stages (Figure 1). The discrepancy may be due to GmMYB73 being an upstream regulator and hence being expressed early in seed development in order to regulate downstream genes including *GL2*, *PLD* and possible other genes. At later stage, the *GmMYB73* is reduced and the downstream factors and lipid biosynthesis genes are activated for lipid accumulation. Another gene *GmbZIP123*, with an increase in expression during soybean seed development, increases lipid contents in seeds of transgenic Arabidopsis plants through promotion of sugar translocation by activation of sucrose transporter genes and cell-wall invertase genes [68].

## Conclusions

Taken together, we find that GmMYB73 promotes lipid accumulation in transgenic plants, possibly through suppression of *GL2* and release of GL2-inhibited *PLDα1* expression. Seed size and thousand-seed weights are also elevated by *GmMYB73* expression in transgenic Arabidopsis plants. Manipulation of *GmMYB73* or the homologues may improve oil production in legume crop plants.

## Methods

### Plant materials

Soybean plants (*Glycine max.* L, cultivar HN44) were grown in field and developing seeds at different stages were collected for RNA analysis. Different organs from three-week-old plants were also harvested for RNA isolation. Arabidopsis *gl2-2* (Col ecotype) and GL2::GUS line [48,69] were used in this study. Double mutant *try cpc* was generated from T-DNA insertion mutant *cpc* (CS6399) and *try* (CS6518) in Columbia (Col) ecotype background and used. The *try cpc*/*GmMYB73* line was generated by crossing *try cpc* with *GmMYB73-*overexpressing line OE-5. *GL2::GUS/GmMYB73* line was generated by crossing GL2::GUS line with *GmMYB73-*overexpressing OE-5. GUS staining and activity measurement were performed as described [59]. The *pldα1* mutant was kindly provided by Prof Xueming Wang (University of Missouri). Arabidopsis plants were grown under standard conditions [60].

### *GmMYB73* gene cloning and plant transformation

*GmMYB73* gene was amplified from leaf cDNAs using gene-specific primers (5′-GGATCCATGGCTGACATAGATC-3′) and 5′-GGTACCTTGGCTAGT CGAAAATC-3′) with BamHI and KpnI restriction site, and cloned into pPROKII. The pPROKII-*GmMYB73* was transfected into Agrobacterium tumefaciens GV3101 and further introduced into Arabidopsis using floral dip method [70]. Homozygous Arabidopsis transgenic lines were used for further analysis. The construct was also used for transformation of Lotus japonicus (Leo) plants [44] and the seeds from the T1 transgenic plants were collected and subjected to total lipid and fatty acid analysis. The *GmMYB73*-containing vector was also introduced into Agrobacterium rhizogenes strain K599 and the transfected bacterium was used for root infections through injection of soybean (Kefeng No. 1) hypocotyls based on previous and our own protocols [42,71]. Hairy roots were generated at infection sites 14 d after infection and the seedlings were immersed in water for 3 d and then the original main roots were removed. The seedlings with transgenic hairy roots were grown in water for 7 d and then the roots were collected for gene expression and fatty acid analysis.

### Gene expression analysis

Total RNA was extracted using TRNzol reagent (Tiangen), and used for first-strand cDNA synthesis. These cDNAs were subjected to real-time quantitative PCR using SYBR Green Master Mix. All primers were used at a concentration of 10 μM. *GmMYB73*, *PLDα1* and *GmDof4* expression in transgenic plants was examined by real-time quantitative PCR. The qPCR was performed in the Roche LightCycle 480 II system, as follows: precycling steps of 95°C for 2 min and then followed by 40 cycles of 95°C for 15 sec, 58°C for 20 sec and 72°C for 30 sec. *AtACTIN2* (NM_112764.3) and *GmTubulin* (XM_003520891) were used as the gene of reference in Arabidopsis and soybean, respectively.

### Total Lipid and fatty acid analysis

Seeds (10 mg) from each homozygous transgenic Arabidopsis line or leaves (100 mg fresh weight) from heterozygous transgenic Lotus line were used for fatty acid extraction as described [72]. Arabidopsis leaves (20 mg dry weight) were extracted for fatty acids following the description [73]. Soybean transgenic hairy roots (100 mg) were also similarly analyzed. Seed total lipid was quantified using the hexane extraction method as described [18]. Four biological replicate samples from each line were extracted for total lipid and fatty acid analysis. The fatty acids were analyzed by gas chromatography (GC2014, SHIMADZU). The machine was equipped with a 30 m (length) × 0.32 mm (inner diameter) × 0.25 μm (liquid membrane thickness) column (Cat. no. 12498, RESTEK). The initial temperature was maintained at 170°C for 5 min, then increased by 2°C min^-1^ to 210°C. After the run, peaks corresponding to each FA species were identified by FAME analytical standard (Cat. no. 18920-1AMP, Supelco). Concentrations of each sample were normalized against the internal control methyl heptadecanoate. Different batches and/or different generations of the materials were used for lipid analysis and the results were consistent. One set of the results was presented.

### Yeast two-hybrid and one-hybrid assays

Vectors and yeast strains were from Clontech (Matchmake Gold Two-Hybrid System). *GmMYB73* was fused to the sequence encoding GAL4 DNA-binding domain in pGBKT7. *GL3* and *EGL3* were fused with sequence encoding GAL4 activation domain in pGADT7. Protein interaction was revealed by growth of transformants and blue color on media that lacked essential amino acids and contained additional supplements Aureobasidin A and X-a-Gal as substrate.

For yeast one-hybrid, the sequence encoding GL2 HD-ZIP domain (amino acids 1 to 236) was cloned into pGADT7 (pAD) to generate pAD-GL2. Five DNA fragments (1, -1 to -263 bp; 2, -246 to -492 bp; 3, -472 to -753 bp; 4, -739 to -1038 bp; 5, -1021 to -1328 bp) of Arabidopsis *PLDα1* promoter were constructed into pAbAi respectively. Transformation of Y1HGold yeast strain with these pAbAi vectors resulted in bait yeast strains, and these bait cells were further transfected with pAD-GL2. Growth of transformants on SD/-Leu/+AbA indicated GL2 binding to the DNA fragment. NAC core binding site was used as a negative control [42].

### BiFC assay and subcellular localization

The pSAT1-cEYFP and pSAT1-nEYFP vector [74] were used in BiFC assay. *GmMYB73* was fused with *nEYFP; GL3* and *EGL3* were fused with *cEYPF*. Ten μg of each plasmid was used for protoplast transformation. EYFP fluorescence was observed after 16 h incubation in dark using Leica TCS SP5 confocal microscope.

### Scanning electron microscopy

Seeds, leaves and stems from flowering Arabidopsis plants were fixed with 2% glutaraldehyde in phosphate buffer solution, dehydrated by alcohol series, and dipped in isoamyl acetate overnight. After the process to remove isoamyl acetate liquid in Critical Point Dryer (HCP-2), the samples were scanned using HITACHI S-3000 N.

### Measurement of seed size and thousand-seed weight

Photos were taken for seeds under scanning electron microscope and the seed lengths and seed widths from 20 to 30 seeds were determined using ImageJ program. The ratio of seed length to seed width was calculated using the above data. Thousand-seed weight was derived from three samples and each was determined by weighing 1000 seeds.

### Gel mobility shift assay

The truncated GL2 protein (amino acids 1 to 236) that contained the HD-ZIP domain was purified as GL2-His fusion protein. The DNA region of AtPLDа1-4 (-739 to -1038) was separated into eight segments (Additional file 2). Complementary oligonucleotides were annealed and double strand oligonucleotides were labeled with Dig-UTP in labeling buffer. The labeled oligonucleotides were incubated with 0.5 μg GL2 proteins for 30 min, and unlabeled oligonucleotides were also added as competitor. The protein/DNA complexes were separated and exposed to X-ray film.

### Chromatin immunoprecipitation (ChIP) assay

ChIP assay was conducted according to Wang *et al*. [75]. About 2 g of 10-day-old *try cpc* double mutant (separate root and leaf) and *gl2-2* mutant seedlings were cross-linked using 1% formaldehyde solution. Soluble chromatin was subjected to ChIP with anti-GL2 antibody or without antibody, with incubation on rotating platform at 4°C overnight. Chromatin-antibody complexes were collected on salmon sperm DNA/protein A-agarose (Millipore). DNA-protein cross-links were reversed at 65°C for 4 h, and the DNA was purified and used in PCR reactions. Primer pairs (AGCCCTACACGTTTTTAGTTTCAC and GTCGGGCGCACGATTTGGAT) were used for amplification of the fragment of *AtPLDа1-4. ACTIN2* was also amplified as a control.

### Mass spectrometric analyses

Powdered seed samples were extracted overnight in 900 μL of chloroform: methanol (1:1) at 4°C with 1200 rpm in a thermomixer. To break phase, 500 μL of deionized H_2_O and 300 μL of chloroform were added. Samples were vortexed and then centrifuged at 10 000 rpm for 2 min at 4°C. Lower organic phase was extracted and transferred to a new tube. Second and third extraction were carried out by adding 500 μL of chloroform to the remaining aqueous phase followed by incubation in the thermomixer at 4°C, with 1200 rpm for 4 h, respectively.

Leaf samples were homogenized in 450 μL of chloroform : methanol (1:2) plus 50 μL deionized H_2_O and rinsed with another 450 μL of chloroform : methanol (1:2) plus 50 μL deionized H_2_O. Samples were then incubated in a thermomixer at 4°C with 1200 rpm for 30 min. To break phase, 400 μL of deionized H_2_O and 300 μL of chloroform were added. Samples were vortexed and then centrifuged at 10 000 rpm for 5 min at 4°C. Lower organic phase was extracted and transferred to a new tube. Second extraction was carried out by adding 500 μL of chloroform to the remaining aqueous phase.

Extractions were combined and dried using SpeedValco. Dried lipid extracts were stored at -80°C until further mass spectrometric analyses. All solvents used for extraction were ice-cold. Three biological replicate samples from each line were extracted for lipid content analysis.

Lipids were analyzed using an Agilent 1260 HPLC system coupled with a triple quadrupole/ion trap mass spectrometer (4500 Qtrap; Applied Biosystems). Separation of individual lipid classes of polar lipids by normal phase (NP)-HPLC was carried out using a Phenomenex Luna 3 μm-silica column (internal diameter 150 × 2.0 mm) with the following conditions : mobile phase A (chloroform: methanol:ammonium hydroxide, 89.5:10:0.5) and mobile phase B (chloroform:methanol:ammonium hydroxide:water, 55:39:0.5:5.5). Glycerol lipids [diacylglycerides and triacylglycerides] were analyzed using a modified version of reverse phase (RP)-HPLC/ESI/MS/MS described previously [76]. Briefly, separation of the aforementioned lipids was carried out on a Phenomenex Kinetex 2.6 μm-C18 column (i.d. 4.6 × 100 mm) using an isocratic mobile phase chloroform: methanol: 0.1 M ammonium acetate (100:100:4) at a flow rate of 150 μL/min for 18 min. Individual lipid species were quantified by referencing to spiked internal standards using multiple reaction monitoring (MRM) transitions [76].

### Statistical analysis

The data were analyzed with ANOVA or Student’s *t*-test using SPSS 11.5 (SPSS Inc., USA).

## Availability of supporting data

The data sets supporting the results of this article are included within the article and its additional files.

## Abbreviations

ACCase: acetyl-CoA carboxylase; DAG: diacylglycerol; DGAT: diglyceride acyltransferase; PA: phosphatidic acid; PC: phosphatidylcholine; PLD: Phospholipase D.; TAG: triacylglycerol.

## Competing interests

The authors declare that they have no competing interests.

## Authors’ contributions

YFL conducted experiments and drafted the initial manuscript. QTL repeated and added new experiments. XL, QXS, WKZ, BM, and QL contributed to data analysis. WQM and WGD provided soybean materials. SML and GHS contributed to Mass spectrometric analysis. JSZ and SYC conceived of the study, obtained funding, analyzed data and finished the final manuscript. All authors read and approved the final manuscript.

## Supplementary Material

Additional file 1 **Phenotype of transgenic Arabidopsis plants overexpressing *GmMYB73 *** **in comparison with the *try cpc *** **double mutant.** Click here for file

Additional file 2 **Eight small regions in *AtPLDа1-4 *** **used for GL2 binding analysis.** Click here for file
