# Supplementary material for: Soybean GmMYB73 promotes lipid accumulation in transgenic plants
Source: BMC Plant Biol. 2014 Mar 24;14:73. doi: 10.1186/1471-2229-14-73 (PMC3998039; doi:10.1186/1471-2229-14-73)
Supplement: Additional file 1 — Phenotype of transgenic Arabidopsis plants overexpressing GmMYB73 in comparison with the try cpc double mutant. [file 1471-2229-14-73-S1.pdf]

Additional file 1

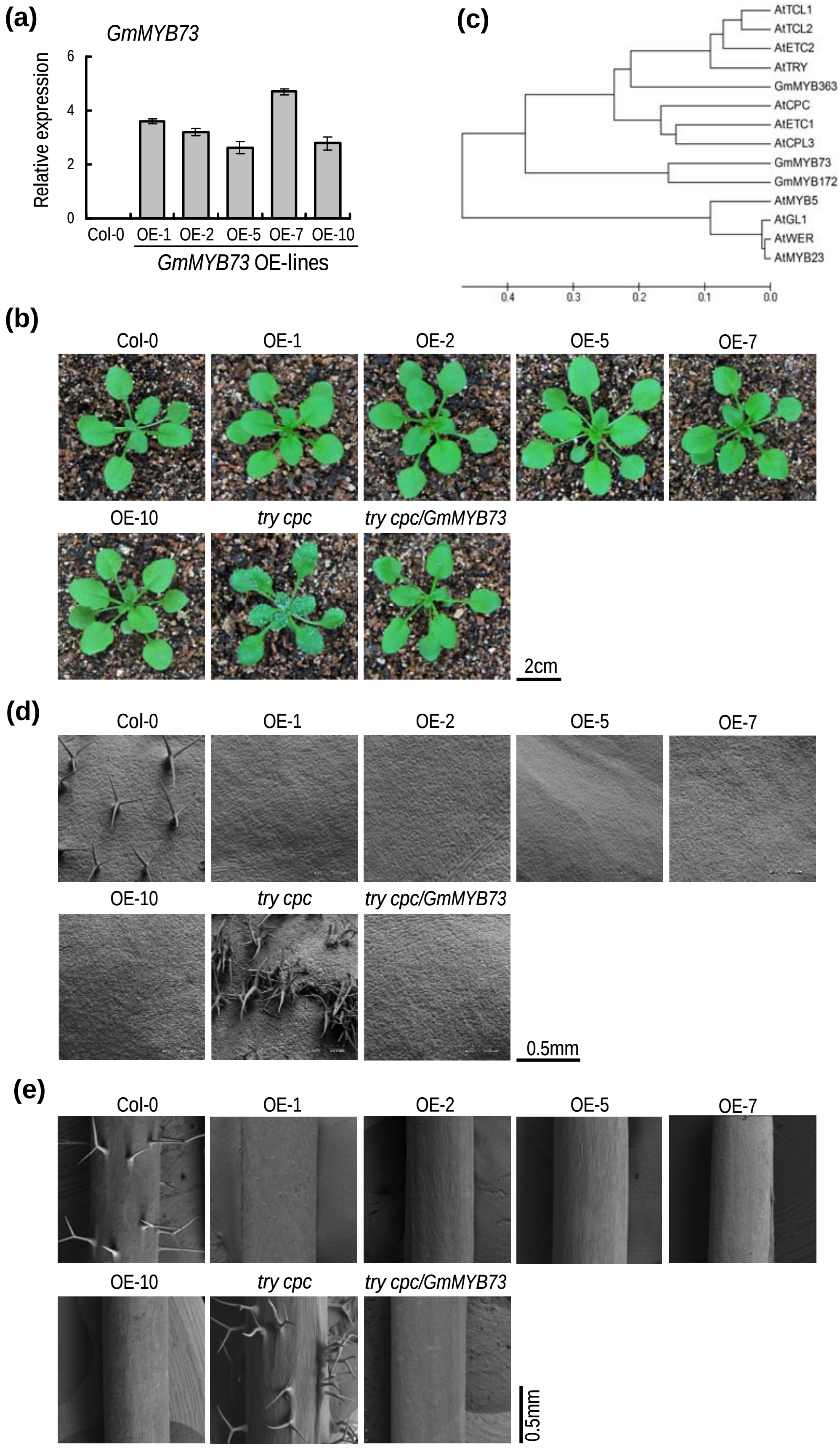

Additional file 1. Phenotype of transgenic Arabidopsis plants overexpressing *GmMYB73* in comparison with the *try cpc* double mutant.
(a) *GmMYB73* expression in leaves of various transgenic lines overexpressing *GmMYB73* (OE-lines).
(b) Inhibition of trichome formation in leaf of different *GmMYB73* OE-lines compared to Col-0. The double mutant *try cpc*, and the line overexpressing *GmMYB73* in the double mutant (*try cpc/GmMYB73*) were included for comparison.
(c) Phylogenetic analysis of GmMYB73 and its homologues in Arabidopsis. The phylogenetic tree was created using MEGA 4.1. The length of each branch represents the distance between the sequences. The bar below the tree indicates the number of nucleotide substitutions per site. GmMYB73 (DQ822927), GmMYB172 (DQ822946) and GmMYB363 (FJ555058) were from soybean and all the other proteins were from Arabidopsis.
(d) Scanning electron microscopy of leaves to show trichome formation. *GmMYB73* suppressed trichome formation on leaves. Others are as in (b).
(e) Trichome formation on stems under scanning electron microscope. Others are as in (b).
