# Supplementary material for: Soybean GmMYB73 promotes lipid accumulation in transgenic plants
Source: BMC Plant Biol. 2014 Mar 24;14:73. doi: 10.1186/1471-2229-14-73 (PMC3998039; doi:10.1186/1471-2229-14-73)
Supplement: Additional file 2 — Eight small regions in AtPLDа1-4 used for GL2 binding analysis. [file 1471-2229-14-73-S2.pdf]

Additional file 2. Eight small regions in *AtPLD α 1-4* used for GL2 binding analysis

AtPLD α 1-4-1	ATAACGATATAATTGTGGTTGGATTATGTTATCTCACATCCTAATAGTAA
AtPLD α 1-4-2	CTAATAGTAATATTTTACGTTACATCTAAAATTATATAATAGAGTGAAAA
AtPLD α 1-4-3	TAGAGTGAAAAAGATAAAATGGAAAGTTTAGTGACTAATTGAAAATCTTG
AtPLD α 1-4-4	GAAAATCTTGAAAAGTTTTAAGAAGGCCGACATAAAAAAAAGGAAATAAAT
AtPLD α 1-4-5	GGAAATAAATATTCAAAGTTGTAATAAAATCCCTGACTTGAGGATAGGGG
AtPLD α 1-4-6	GGATAGGGGAGACAAAAAAAAAAAAAGAGATATCCAAATCGTGCGCCCGAC
AtPLD α 1-4-7	TGCGCCCGACGTTCTGCATCCAAACGTTACGACATCGTTTCCCAAGCTGA
AtPLD α 1-4-8	TCCCAAGCTGAATCCTCACCGTCCATCACAAGAAAACTATA
